# Cannabis: A Toxin-Producing Plant with Potential Therapeutic Uses

**DOI:** 10.3390/toxins13020117

**Published:** 2021-02-05

**Authors:** Zeinab Breijyeh, Buthaina Jubeh, Sabino A. Bufo, Rafik Karaman, Laura Scrano

**Affiliations:** 1 Pharmaceutical Sciences Department, Faculty of Pharmacy, Al-Quds University, Jerusalem Abu Dis P144, Palestine; z88breijyeh@gmail.com (Z.B.); bjubeh@gmail.com (B.J.); 2 Department of Sciences, University of Basilicata, 85100 Potenza, Italy; 3 Department of Geography, Environmental Management & Energy Studies, University of Johannesburg, Johannesburg 2092, South Africa; 4 Department of European Cultures (DICEM), University of Basilicata, 75100 Matera, Italy; laura.scrano@unibas.it

**Keywords:** *Cannabis sativa*, marijuana, hemp, cannabinoids, endocannabinoids, cannabinoid receptors, Δ-9-tetrahydrocannabinol (THC), therapeutics, toxicity, abuse

## Abstract

For thousands of years, *Cannabis sativa* has been utilized as a medicine and for recreational and spiritual purposes. Phytocannabinoids are a family of compounds that are found in the cannabis plant, which is known for its psychotogenic and euphoric effects; the main psychotropic constituent of cannabis is Δ9-tetrahydrocannabinol (Δ9-THC). The pharmacological effects of cannabinoids are a result of interactions between those compounds and cannabinoid receptors, CB1 and CB2, located in many parts of the human body. Cannabis is used as a therapeutic agent for treating pain and emesis. Some cannabinoids are clinically applied for treating chronic pain, particularly cancer and multiple sclerosis-associated pain, for appetite stimulation and anti-emesis in HIV/AIDS and cancer patients, and for spasticity treatment in multiple sclerosis and epilepsy patients. Medical cannabis varies from recreational cannabis in the chemical content of THC and cannabidiol (CBD), modes of administration, and safety. Despite the therapeutic effects of cannabis, exposure to high concentrations of THC, the main compound that is responsible for most of the intoxicating effects experienced by users, could lead to psychological events and adverse effects that affect almost all body systems, such as neurological (dizziness, drowsiness, seizures, coma, and others), ophthalmological (mydriasis and conjunctival hyperemia), cardiovascular (tachycardia and arterial hypertension), and gastrointestinal (nausea, vomiting, and thirst), mainly associated with recreational use. Cannabis toxicity in children is more concerning and can cause serious adverse effects such as acute neurological symptoms (stupor), lethargy, seizures, and even coma. More countries are legalizing the commercial production and sale of cannabis for medicinal use, and some for recreational use as well. Liberalization of cannabis laws has led to increased incidence of toxicity, hyperemesis syndrome, lung disease cardiovascular disease, reduced fertility, tolerance, and dependence with chronic prolonged use. This review focuses on the potential therapeutic effects of cannabis and cannabinoids, as well as the acute and chronic toxic effects of cannabis use on various body systems.

## 1. Introduction

*Cannabis sativa* L. (*C. sativa*) is a flowering, fast-growing, 1–2 m in height, shrub plant belonging to the *Cannabis* genus and Cannabaceae family. *C. sativa* is commonly known as hemp, cannabis, or marijuana, originates from Central Asia, and is widely distributed in temperate and tropical areas [1,2,3,4,5]. There are several preparations of *C. sativa*, which can be smoked by a cigarette or a hash pipe, inhaled, or ingested in the form of candy or brownies; the most common is marijuana (dried, crushed flower tops, stems, and leaves of *C. sativa* plant), and hashish (resins of the flowering tops of *C. sativa* plant) [6,7,8,9].

**Figure 1:**
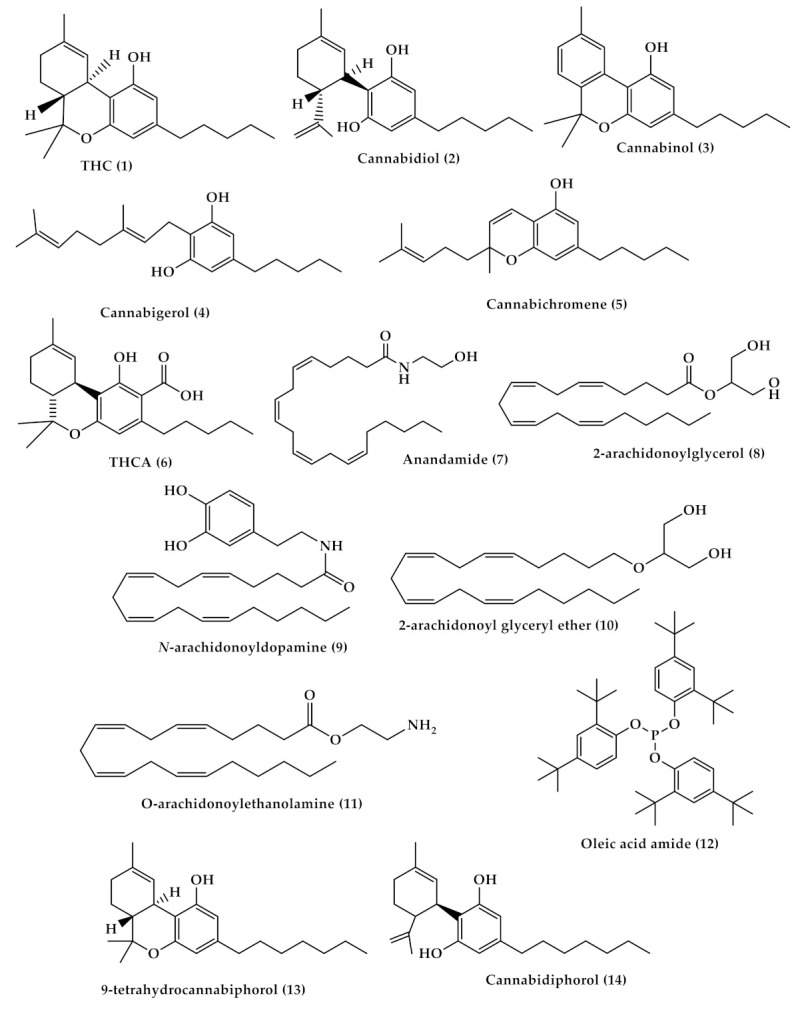
Chemical structures of Δ-9-tetrahydrocannabinol (THC, (**1**)), cannabidiol (**2**), Δ9- cannabinol (**3**), cannabigerol (**4**), cannabichromen (**5**) tetrahydrocannabinol-4-oic acid (THCA, (**6**)), anandamide (AEA, (**7**)) 2-arachidonoylglycerol (2-AG, (**8**)), *N*-arachidonoyldopamine (NADA, (**9**)), 2-arachidonoyl glyceryl ether (2-AGE, (**10**)), *O*-arachidonoylethanolamine (**11**), oleic acid amide (OA, (**12**)), Δ9-tetrahydrocannabiphorol (Δ9-THCP, (**13**)) and cannabidiphorol (CBDP, (**14**)).

The cannabis plant is very rich in phytochemicals; it contains more than 560 known compounds and there are over 120 cannabinoids identified in the literature [1,10,11,12]. Phytocannabinoids are known for their physiological and often psychotogenic effects; out of a large number of cannabinoids present in the cannabis plant, *trans*-Δ-9-tetrahydrocannabinol (THC (1), Figure 1) and cannabidiol (CBD (2), Figure 1), are the most commonly described [10,13], in addition to other minor phytocannabinoids such as cannabinol ((3), Figure 1), cannabigerol ((4), Figure 1), and cannabichromene ((5), Figure 1) [14].

Cannabis is used for recreational purposes in some geographic areas, and at the same time it is categorized as a drug of abuse with strict restrictions in most countries. Nowadays, cannabis legalization is increasing rapidly, with more than 200 million users worldwide, and for this reason it necessitates a greater awareness of its potential benefits and harms. According to the United Nations Office on Drugs and Crime (UNODC), *C. sativa* is the most popular illicit drug of the 21st century. A dilemma surrounding cannabis safety and potential therapeutic effectiveness arises among researchers because most of the systemic reviews on cannabis use reported that cannabis has harmful outcomes and showed the clinical features of acute cannabis ingestion among children and adults that include anxiety, respiratory distress, decreased levels of consciousness, confusion and intoxication, psychiatric symptoms, and gastrointestinal adverse effects, especially among adults. Other reviews reported insufficient evidence of harm, or no evidence of harm outcomes, and encouraged cannabis legalization, showing its potential medical and therapeutic effects in treating different medical disorders such as cancer, neurological conditions, and others [15,16,17,18,19,20]. Therefore, it is important to better understand this plant and give proper knowledge to highlight the need for new guidelines and policies to regulate cannabis use. This review gives a brief summary about chemistry, pharmacology, and the potential therapeutic uses of cannabis; in addition, it discusses the effect of acute and long-term use of cannabis and its toxicity profiles.

## 2. Cannabis Chemistry and Pharmacology

**Figure 2:**
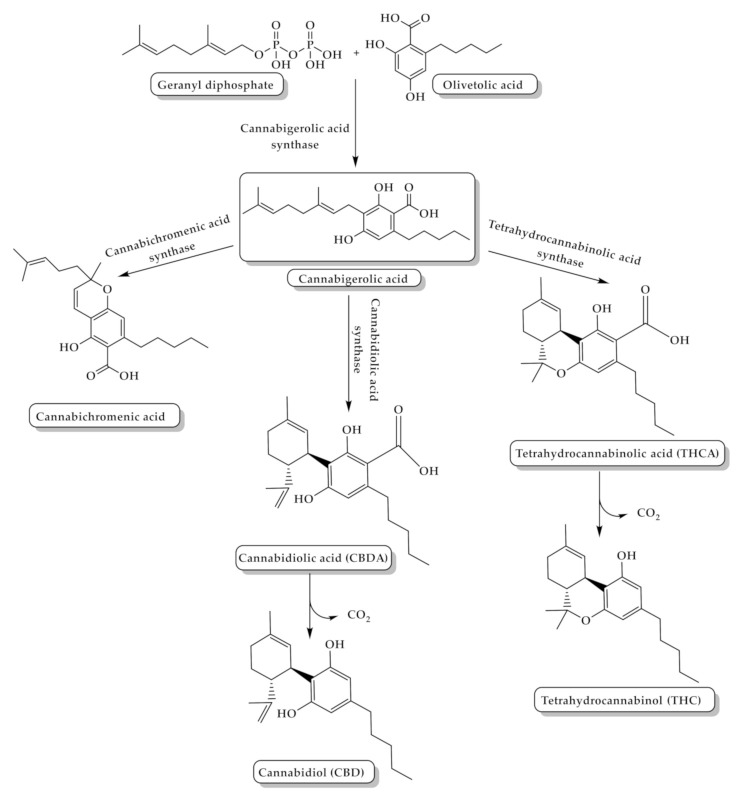
The biosynthesis pathway of tetrahydrocannabinol (THC) and cannabidiol (CBD) from the coupling of olivetolic acid and geranyl diphosphate.

Cannabinoids can be classified into three groups according to their source of production: phytocannabinoids, endocannabinoids, and synthetic cannabinoids. The major cannabis chemical constituents are phytocannabinoids, which comprise a group of C21 terpene phenolic compounds, or C22 for the carboxylate forms, predominantly produced in cannabis. The plant also contains a wide range of non-cannabinoid terpenes and phenolic compounds [14,21]. Biosynthesis of phytocannabinoids is achieved by the coupling of olivetolic acid and geranyl diphosphate to produce different cannabinoids (Figure 2) [21].

Phytocannabinoids are biosynthesized in carboxylated form and can be decarboxylated by heat [22]. THC is the main and most potent psychoactive compound in cannabis that is responsible for the intoxicating effect which causes “highness” on consumption, and has a medical effect in which it can be used as an antiemetic and anti-inflammatory, in addition to its ability to reduce neuropathic and chronic pain [23]. The potency of cannabis products is determined by their THC content [24]. Δ9-tetrahydrocannabinol-4-oic acid (THCA (**6**), Figure 1) is the acidic precursor of THC and the major constituent of drug-type cannabis. THCA undergoes decarboxylation to THC by the heat of combustion during smoking [3]. On the other hand, CBD is a non-intoxicating THC isomer with no psychotogenic effects, but it shows various pharmacological activities, such as pain and spasticity control [25].

**Figure 3:**
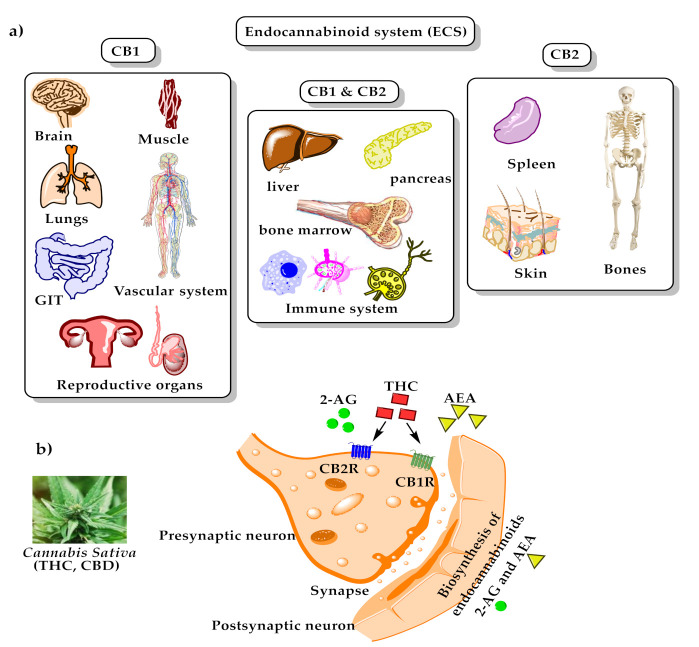
The endocannabinoid system; (a) distribution of endocannabinoids receptors through human body; (b) binding of endogenous anandamide (AEA), 2-arachidonoylsn-glycerol (2-AG), and exogenous Δ^9^-tetrahydrocannabinol (Δ^9^-THC) with cannabinoid receptors type 1 (CB1) and type 2 (CB2).

In the body, phytocannabinoids bind to specific receptors distributed throughout the body, which are called endocannabinoid receptors. Endocannabinoid receptors, along with their endogenous neurotransmitters N-arachidonoylethanolamine (anandamide, AEA) ((7), Figure 1) and 2-arachidonoylglycerol (2-AG) ((8), Figure 1), and the enzymes that are responsible for endocannabinoids synthesis and degradation, form the endocannabinoid system (ECS) [26]. Recently, other molecules have also been considered as endocannabinoids, such as *N*-arachidonoyldopamine (NADA, (9), Figure 1), 2-arachidonoyl glyceryl ether (2-AGE, (10), Figure 1), *O*-arachidonoylethanolamine ((11), Figure 1), and oleic acid amide (OA, (12), Figure 1) [27]. There are two endocannabinoid receptors, CB1 and CB2, which belong to the G-protein coupled receptors family and are found in the immune tissues (where CB2 is mainly expressed) and in the central nervous system (where CB1 is mainly expressed and, hence, mediates the psychoactive effects of *Cannabis*). Both exert inhibitory neuronal activity upon their interactions with the cannabinoids [28,29]. AEA has a high affinity for CB1 compared to CB2, while 2-AG has moderate affinity for both CB1 and CB2. Exogenous THC is a partial CB1 and CB2 agonist, while phytocannabinoid CBD has a low affinity for these receptors [29,30]. Recently, two phytocannabinoids were discovered and isolated from *Cannabis sativa*: Δ9-tetrahydrocannabiphorol (Δ9-THCP, (13), Figure 1), a Δ9-THC homolog that binds with high affinity to both CB1 and CB2, and cannabidiphorol (CBDP, (14), Figure 1), a CBD homolog that has no available reports on its pharmacological effects yet [31]. For the binding mode of some of the mentioned compounds with cannabinoid receptors type 1 (CB1) and type 2 (CB2), please see Figure 3.

**Figure 4:**
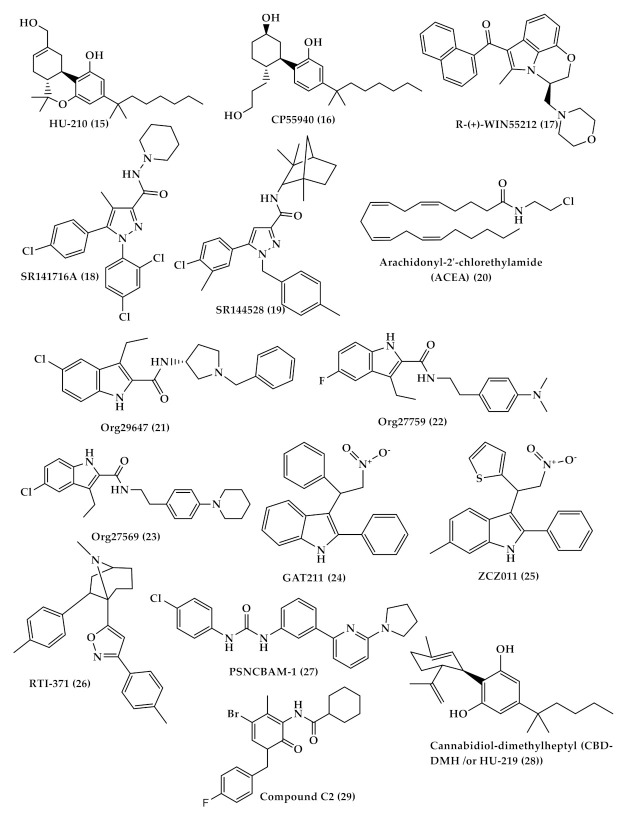
Chemical structures of some synthetic agonist and antagonist cannabinoid; (−)-11-hydroxy-D8--THC dimethyl heptyl (HU-210) (**15**), CP55940 (**16**), R-(+)-WIN55212 (**17**), SR141716A (**18**), SR144528 (**19**), arachidonyl-2′-chlorethylamide (ACEA) (**20**), and allosteric modulators; Org29647 (**21**), Org27759 (**22**), and Org27569 (**23**), GAT211 (**24**), ZCZ011 (**25**), RTI-371 (**26**) and PSNCBAM-1 (**27**), cannabidiol-dimethylheptyl (CBD-DMH /or HU-219 (**28**)) and compound C2 (**29**).

Several cannabinoid receptor agonists bind more or to a lesser extent to CB1 and CB2 receptors such as classical (THC and (−)-11-hydroxy-D8--THC dimethyl heptyl (HU-210) ((15), Figure 4)), nonclassical (CP55940 (16), Figure 4)), aminoalkylindole (R-(+)-WIN55212 ((17), Figure 4)) which has a higher affinity to CB2 more than CB1 receptor) [32], and antagonist-inverse agonists (SR141716A ((18), Figure 4) for CB1 and SR144528 ((19), Figure 4) for CB2) [33]. In general, many antagonists show high selectivity toward the CB1 receptor which allows differentiation between CB1 and CB2, while numerous agonists show low selectivity between cannabinoid receptors. Despite that, some agonists, such as arachidonyl-2′-chlorethylamide (ACEA (20), Figure 4) compound, show CB1 high selectivity [33,34,35]. In addition, allosteric modulators that bind to different sites other than the ligand orthosteric site affect the stimulus of cannabinoid receptor by either enhancing or reducing its activity. Three allosteric modulators of CB1 were first described by Price et al.—Org29647 (21), Org27759 (22), and Org27569 (23) (Figure 4)—which showed an enhancement of CP-55940 agonist binding to CB1 and reduction in SR141716 antagonist/inverse agonist binding [36]. Other synthetic CB1 allosteric modulators were also developed, such as GAT211 (24), ZCZ011 (25), RTI-371 (26) and PSNCBAM-1 (27) (Figure 4) [37]. On the other hand, there are just two synthetic allosteric modulators of CB2 cannabidiol-dimethylheptyl (CBD-DMH /or HU-219 (28)) and compound C2 (29) (Figure 4) [38,39].

Cannabinoids (both phytocannabinoids and endogenous) also interact with receptors other than cannabinoid receptors and act simultaneously on multiple targets within the nervous system, such as putative non-CB1/CB2 cannabinoid G protein-coupled receptor (GPCR) 55 [40], transient receptor potential (TRP) channels, the N-arachidonoyl glycine (NAGly) receptor [41,42], and 5- hydroxytryptamine (5-HT) receptors [43,44].

## 3. Cannabis and Cannabinoids: Potential Therapeutic Uses

For thousands of years, the cannabis plant has been utilized in traditional remedies. Cannabis-induced analgesia is the most common reason for using cannabis medically. During the past several decades, synthetic chemical analogues of THC were made and are known as synthetic cannabinoids. Several innovative drugs, structurally similar to or mimicking the activity of THC and CBD, have been developed and applied clinically, or are still in clinical studies, for treating conditions like chronic pain, epilepsy, and multiple sclerosis, and for use as appetite stimulants and antiemetic agents in HIV/AIDS and cancer patients [45,46,47]. Therefore, cannabinoids can provide a lead compound for new drug development.

Treatment with herbal cannabis and cannabis-based medicine can be administered in various routes, such as smoking, vaporization, oral, oromucosal, and others; which affect the absorption and toxicity of cannabinoids. Long-acting oral preparations are the mainstay of treatment for chronic conditions, and vaporization can be utilized as an add-on treatment for acute symptoms. Adverse events of cannabis are mainly attributed to THC; therefore, the total daily dose-equivalent of THC should generally be limited to 30 mg/day or less, preferably combined with CBD [48]. In contrast to medical cannabis, recreational cannabis tends to be high in THC content to attain the euphoric high. In medical cannabis, some strategies should be applied to minimize the adverse effects of cannabis. Adverse effects of THC, such as fatigue, tachycardia, and dizziness, are avoided by starting with low doses and slow titration of cannabis preparations. The general approach of cannabis medical prescription is ‘start low, go slow, and stay low’. In addition, combining CBD with THC can balance THC side effects [48,49]. Medical cannabis patients frequently use CBD-predominant products with the minimum amounts of THC to have the best improvement in symptoms and quality of life, with the fewest adverse effects.

### 3.1. Pain Treatment with Cannabis 

Chronic pain (cancer-related pain, neuropathic pain, or pain associated with multiple sclerosis) is a major health issue, especially in older people. In most cases, chronic pain is treated with opiates (which may lead to drug abuse), antidepressants, and anticonvulsant drugs [50]. Currently, the only analgesics to treat severe pain are opioids, but they are associated with unpleasant side effects such as sedation, persistent constipation, loss of appetite, nausea, respiratory depression, tolerance, dependence, and opioid-induced hyperalgesia, which limit their clinical use [51].

Due to opioids’ drawbacks, cannabis is being considered as a useful treatment for pain, and its legalization in some countries led to a decrease in opioid overdose deaths [52]. According to some surveys, healthcare providers in general believe in the benefits of cannabis to patients and can facilitate their access to medical cannabis [53]. In addition, the majority of patients believe that cannabis is useful in treating pain and as a replacement for opioid medication, and others believe in cannabis safety and efficacy to justify cannabis use for different medical conditions. Other patients may also use cannabis alongside prescription drugs, hence making the efficacy of cannabis on well-being unknown [53,54,55]. On the other hand, several systematic reviews showed that cannabinoid use for treating chronic pain has moderate-quality evidence and most of the individual studies did not reach statistical significance. In addition, other studies showed no evidence that cannabis use reduced pain severity or opiate use in people with chronic pain, which indicates the importance for more and large, well-designed clinical trials that include people with different complex comorbidities to determine the efficacy of cannabis use for chronic pain [56,57].

Nowadays, cannabinoids are being widely explored for different types of pain (nociceptive, neuropathic, and central). Injured tissues of neural and non-neural cells produce endocannabinoids such as anandamide, and 2-AG, which modulate pain signals through the activation of cannabinoid receptors. CB1 receptors are abundant in nociceptive and non-nociceptive sensory neurons of the dorsal root ganglion (DRG), the spinal cord, the brain, mast cells, macrophage defense cells, the trigeminal ganglion (TG), and epidermal keratinocytes. Few CB2 receptors are expressed in these regions, but they are increased when there is peripheral nerve damage. The study of the mode by which endogenous cannabinoids exert their effects is crucial for comprehending the efficacy of exogenous cannabinoids for pain treatment [58].

Cannabinoids control pain by acting on several receptors by different mechanisms; for example, THC has the ability to (1) inhibit prostaglandin E-2 synthesis and stimulate lipoxygenase [59], (2) decrease 5-hydroxytryptamine (5-HT) release from platelets and its synaptosomal uptake while increasing its cerebral production [50,60], (3) affect the trigeminovascular system in migraines [61], (4) alter dopaminergic function [62], (5) inhibit pre-synaptically glutamate release [63], and (6) activate vanilloid-transient receptor potential-2 (TRPV2) mainly and moderately modulate TRPV3, TRPV4, TRPA1 (ankyrin 1), and TRPM8 (melastatin 8), with no reported modulation of TRPV1 [64]. On the other hand, CBD is a vanilloid-transient receptor potential-1 (TRPV-1) or capsaicin receptor agonist that is capable of inhibiting fatty-acid amide hydrolase enzyme (FAAH), which is responsible for the hydrolysis of anandamide and inhibits its reuptake. Moreover, CBD has the ability to inhibit the hepatic metabolism of THC to 11-hydroxy-THC, a more psychoactive compound, and increases its half-life, and as a result, reduces its side effects [59]. CBD may improve anti-inflammatory effects by (i) decreasing reactive oxygen species (ROS), tumor necrosis factor (TNF-α) levels, and pro-inflammatory cytokines; (ii) inducing T cell apoptosis; (iii) inhibiting T cell proliferation; and (iv) reducing migration and adhesion of immune cells, which all result in a reduction in oxidative stress and inflammation [65,66,67]. CBD-mediated inflammatory suppression is attributed to either CB1 or CB2 receptors. Although CBD has weak affinity to CB1 and CB2, CBD inhibits FAAH and elevates anandamide, an endogenous cannabinoid which exhibits affinity for CB1and CB2 receptors. Additionally, CBD has immune effects mediated through a blockade of GPR55 receptors, and activation of TRPV1, adenosine A2A, and PPAR-c receptors [68]. These and many other mechanisms have led to considering CBD as an endocannabinoid modulator, which made pharmaceutical companies develop new chemical entities (NCEs) that mimic its action. In addition, cannabichromene (CBC) also has anti-inflammatory and analgesic effects, but its effects are weaker than THC’s effects. Moreover, cannabigerol (CBG) inhibits GABA uptake and is considered as a more potent analgesic than THC. So, CBG can be used as a muscle relaxant in spasticity [50]. Other cannabinoids are also used as analgesics to treat pain and are listed in Table 1.

**Table 1:** Cannabinoids used as analgesics for pain treatment.

Analgesic Cannabinoids	Theraputic Actions
Dronabinol (Marinol (30), Figure 5)	A synthetic oral form of THC and a partial agonist at the CB1 receptor. Approved in the USA in 1985 for nausea associated with chemotherapy and for appetite stimulation in HIV/AIDS [69]. Used for treating the pain of patients with multiple sclerosis. [70].
Nabilone (Cesamet (31), Figure 5)	A synthetic orally administrated dimethyl heptyl analog of THC [71]. Approved in 1981 by the US FDA for the treatment of nausea and vomiting induced by chemotherapy [72], and is reported to be dispensed off-label for the management of pain [73], and treatment of fibromyalgia in some studies [74,75].
Ajulemic acid (AJA (32), Figure 5)	An oral synthetic non-intoxicating analog of THC. Stimulate endogenous eicosanoids that limit inflammation progress and resolve fibrosis [76,77]. AJA is still under study to evaluate its effect and toxicity [76,78].
Sativex	A cannabis-based oromucosal spray containing a mixture of a 1:1 ratio of THC and CBD. Approved in several European countries. Used as an add-on therapy of multiple sclerosis (MS) related spasticity in patients non-responsive to conventional anti-spastic therapies [79].
WIN-55,212-2 ((R)-(+)-[2,3-Dihydro-5-methyl-3[(4-morpholinyl)methyl]pyrrolo[1,2,3-de]-1,4-benzoxazinyl]-(1-naphthalenyl)methanone) (WIN (17), Figure 4)	A synthetic cannabinoid that binds non-selectively to CB1 and CB2 receptors Has the ability to alleviate neuropathic pain by suppressing mechanical allodynia and thermal hyperalgesia [80,81].
Selective CB2 agonists (HU-308, AM1241, JWH-133 and GW405833);	HU308 (4-[4-(1,1-diemethylheptyl)-2,6-dimethoxyphenyl]-6,6-dimethyl-bicyclo[3.1.1]hept-2-ene-2-methanol (33), Figure 5) binds selectively to CB2 and its agonist activity results in peripheral antihyperalgesic and anti-inflammatory effects [82]. AM1241 (2-iodo-5-nitro-phenyl)-[1-(1-methyl-piperidin-2-ylmethyl)-1*H*-indol-3-yl]-methanone (34), Figure 5) acts by producing peripheral-mediated antinociception, inducing CB2-mediated antihyperalgesic effects and stimulating the release of β-endorphin from skin keratinocytes [82]. JWH-133 ((6aR,10aR)-3-(1,1-dimethylbutyl)-6a,7,10,10a-tetrahydro-6,6,9-trimethyl-6*H*-dibenzo[b,d]pyran (35), Figure 5) inhibits inflammatory and neuropathic hyperalgesia [82]. Another CB2 selective agonist form called JWH015 is given intrathecal for the treatment of bone cancer pain where it has the ability to downregulate IL-1β and IL-6 [83]. GW405833 (2,3-dichloro-phenyl)-[5-methoxy-2-methyl-3-(2-morpholin-4-yl-ethyl)-indol-1-yl]-methanone) ((36), Figure 5) [82] also has an antihyperalgesic effect and reduces allodynia in inflammatory pain [84].

### 3.2. Cannabis as an Anti-Emetic and Appetite Stimulant

Emesis (vomiting) is a protective mechanism to prevent the digestion of harmful substances, and at the same time, it can be considered as a symptom of a disease or a side effect of specific medications such as cancer chemotherapeutic agents [85]. Blockage of 5-hydroxytryptamine subtype, 5-HT3, receptor was found to suppress acute emetic response induced by the chemotherapeutic agent cisplatin [86]. Many 5-HT3 antagonists have been developed but showed low effectiveness in suppressing acute nausea and reducing delayed nausea and vomiting [87]. The cannabis plant has been used for several centuries for treating nausea and vomiting, and in clinical trials, it was found to be an effective antiemetic agent [85]. Cannabinoids act as antiemetic agents by interacting with CB1 and 5-HT3 receptors that are located centrally and in the dorsal vagal complex (DVC), where emesis is activated. Anandamide, THC, and different synthetic cannabinoids were tested on animal models and showed to have an allosteric inhibitor effect on 5-HT3 receptors in the DVC, in addition to the activation of CB1 receptors, which reduces emesis [88,89]. 

Furthermore, *C. sativa* was found to be able to stimulate appetite (hyperphagia) [90]. Several studies have shown that after cannabis smoke inhalation, an increase in daily caloric intake and food consumption was observed [91]. Dronabinol was evaluated for short- and long-term clinical trials for the treatment of malnutrition and wasting syndrome in HIV-infected patients and was associated with an increase in appetite and body fat by 1%, by the activation of CB1 receptors in the hypothalamus [92,93]. Non-intoxicating cannabigerol (CBG) was also investigated and found to have the ability to penetrate the blood–brain barrier and interact with endocannabinoid and non-endocannabinoid targets, which are involved in the control of feeding and energy balance, and hence mediates the stimulation of feeding behavior [90].

Cannabinoids are useful in the treatment of anorexia and chemotherapy-induced nausea and vomiting, despite their low potency compared to other available antiemetics. However, cannabinoids are the only antiemetic that increases appetite [94]. Further studies are needed to support the promising effect of cannabinoids as an appetite stimulator and for treating nausea.

### 3.3. Cannabis and Multiple Sclerosis (MS)

Patients with MS use various cannabinoid compounds (either over the counter or illegally) for different indications, such as spasticity, relaxation, tremors, pain, sleep, and anxiety. The anti-inflammatory effect of cannabinoids may help in suppressing the disease activity in multiple sclerosis by reducing inflammatory factors. In addition, cannabinoids such as Sativex (nabiximols) were approved in several countries for the treatment of severe spasticity in MS by acting at the presynaptic CB1 receptors, which in response reduces massive glutamate release and regulates glutamatergic excitability during spasticity [95]. Recent studies showed that 20–60% of MS patients are currently using cannabis, and 50–90% are willing to consider its usage if it was legal. Therefore, further research is needed to investigate cannabis’ effects in MS patients and to answer several questions related to cannabis use, dosage, long-term effects and more [96].

### 3.4. Cancer Treatment by Cannabis 

Cannabinoids are primarily used in cancer for palliative care to alleviate pain, stimulate appetite, and relieve nausea. In recent years, more studies have been done on the potential use of cannabinoids as antitumor and symptomatic relief agents in cancer patients. The role of the endocannabinoid system is not completely clear in cancer, but several studies suggest that cannabinoid receptors and endogenous ligands are overexpressed in tumor tissue [97]. Cannabinoids have been found to inhibit tumor cell proliferation, angiogenesis, tumor invasion, and induce apoptosis in vitro and in vivo by the activation of cannabinoid receptors [98]. In other supportive studies, it was observed that the deletion or inhibition of CB1 receptors resulted in the acceleration of intestinal adenoma growth in genetic mice colon cancer models, while the activation of CB1 receptors induced tumor cell death by the downregulation of an anti-apoptotic survival factor [99]. The known mechanisms of cannabinoids that induce apoptosis are an interaction with orphan G-protein coupled receptor 55 (GPR55), transient receptor potential channel subfamily V member 1 (TRPV1) [100], and transient receptor potential channel subfamily M member 8 (TRPM8), like CBD and cannabigerol which act as potent antagonists for TRPM8 receptors [101]. In addition, CB2 receptors (CB2R) have immunomodulatory effect where their activation can affect cytokine release from immune cells, inhibition of IFN-γ production and suppression of T-cell proliferation [102]. Moreover, cannabinoids have the ability to block the vascular endothelial growth factor (VEGF) pathway and activate VEGFR-2 by blocking ceramide biosynthesis which can result in angiogenesis inhibition [103]. Further studies are needed to support the idea of introducing cannabinoids as anticancer agents [104].

## 4. Cannabis Poisoning 

Cannabinoid use as analgesics, antiemetics, appetite stimulants, and anti-spastics are already approved by regulatory bodies such as the US FDA, Health Canada, and the European Medicines Agency. However, several undesired effects may be associated with cannabis and synthetic cannabinoid (SC) use, such as psychosis and self-harm. Exposure to high concentrations of THC could lead to psychological and neurological events, such as dizziness, drowsiness, ataxia, seizures, hypotonia, stupor, coma, and ocular features such as mydriasis and conjunctival hyperemia, in addition to gastrointestinal disorders, and cardiovascular features such as tachycardia, arterial hypertension, and postural hypotension [105,106,107,108,109,110,111,112,113]. However, the use of SCs can lead to more toxic side effects, which may be attributed to the low or no CBD content that has a protective role (anxiolytic and antipsychotic properties), and/or to the high affinity to CB1 receptors compared to THC. The use of SCs is associated with typical acute adverse effect (ex. euphoria, delusions, anxiety, panic attacks, vomiting, seizures, dizziness and others), cardiovascular side effects (tachycardia and hypertension), and long-term adverse effects (high abuse, dependence and tolerance) [111,113]. On the other hand, recreational use of cannabis is considered of low harm, but at the same time may cause damage to the physical and mental health of users in short- and long-term use. Several studies showed that medical cannabis users employed it for recreational reasons, and this may result in intolerable psychoactive effects in patients with no recreational experience, leading them to discontinue medical use [114,115]. Smoking dried cannabis leaves is the preferred consumption method for recreational use because it affords high bioavailability and easy preparation and dosing. Most users experience relaxation, mild euphoria, time distortion with little dysphoria, and anxiety effects, which can increase with the amplification of the THC content, especially in naive users [116].

Cannabis intoxication is dose-related, and its absorbance depends on the route of administration and concentration being used. Inhaled doses of 2–3 mg and ingested doses of 5–20 mg of THC can affect memory and cause short-term memory impairment and loss of attention, while inhaled doses more than 7.5 mg/m2 in adults and oral doses of 5–300 mg in pediatric subjects can cause more serious effects, such as respiratory depression, panic, anxiety, hypotension, myoclonic jerking and other symptoms [117]. The LD50 (the lethal dose at which 50% of the sample population dies) of THC is not determined in humans due to ethical reasons, but in animals it ranges from 40 to 130 mg/kg intravenously. The LD50 of THC inhalation from smoked cannabis in Fisher rats is 42 mg/Kg, a value that is similar to the IV vascular access port value, which indicates that THC is the active intoxicant of smoked cannabis [117,118].

Cannabis-induced adverse effects may be influenced by other several factors such as genetic variation, age, sex, ethnicity, and duration and frequency of cannabis use [119,120,121]. Significant toxicity from cannabis and cannabinoid-containing substances is uncommon in adults, unlike children, who may develop significant symptoms. Fortunately, these toxicity symptoms are usually short-lived and last for several hours [122,123]. A case of cannabis poisoning was reported in a preschool child who was on oral hemp seed oil for three weeks after medical prescription to strengthen his immune system. The child exhibited acute neurological symptoms such as stupor and low reactivity to stimulation, associated with conjunctival hyperemia. THC was detected in the product ingested by the child, and the acid metabolite of THC was detected in the child’s urine [124]. Carstairs et al. reported a case of a 14-month-old child who ingested hashish and was in a prolonged coma for more than 48 hours. The THC metabolite, 11-nor-carboxy-Δ9–THC, was detected in high levels in the patient’s urine, and the patient’s clinical improvement coincided with the decline of the THC metabolite’s level in urine [125]. 

Generally, cannabis has modest harm that can be avoidable if the starting dose is low, and dose titration is slow. Medical use of cannabis may be associated with adverse events, like drowsiness, fatigue, dizziness, dry mouth, anxiety, nausea, cognitive effects, euphoria, blurred vision, headache, and orthostatic hypotension. Smoking medical cannabis is commonly associated with coughing, phlegm, and bronchitis [126,127,128]. Recreational use is done with no calculated doses nor medical monitoring, likely to be heavy and sustained, and is done most commonly by harmful methods like smoking. Hence, recreational cannabis users are more prone to cannabis poisoning and toxicity. The following discusses harm related to nonmedical cannabis use.

### 4.1. Cannabis’ Effects on the Cardiovascular System

Cannabinoids’ psychological and physiological side effects are well reported, but there is little awareness about the potential cardiovascular effects that are associated with cannabis use. Cannabis can induce higher heart rates, blood pressure, venous carboxy-hemoglobin levels, and can trigger arrhythmias and myocardial infarctions [129]. Cannabinoids’ impact on cardiac function is hypothesized to be a result of stimulation of CB1 receptors. The CB1 receptors are present in the heart, and their stimulation can lead to acute tachycardia and bradycardia, hypotension, decreased cardiac contractility chronically, and elevated oxidative stress [130,131]. Cannabis causes systemic vasodilation, orthostatic hypotension, and an acute dose-dependent increase in the heart rate as well. Some of these effects are mediated by the autonomic nervous system [132]. A study by Wagner et al. found that endocannabinoids can mediate hypotension in experimental models of myocardial infarction [133], similarly to that observed with the use of exogenous cannabinoids. Generally, cannabis-induced cardiovascular symptoms are well tolerated in most young healthy people, but this is not the same for patients with established coronary or atherosclerotic diseases [132,134]. Bachs and Mørland [135] reported six cases of sudden death in males aged 17–43 years old possibly related to cannabis ingestion. Five cases had no previous heart disease, with a record of illicit drug use in just two cases. The sixth case had a previous coronary heart condition and was using heart medications. In all cases, the probable cause of death was an acute cardiovascular event and the presence of cannabis alone was detected in the blood analyses, indicating recent cannabis intake. Reports have shown that the deceased individuals seemed to be occasional cannabis users and not heavy drug addicts. 

Cannabis exposure can increase the risk of myocardial infarction five-fold in the hour after smoking and declined rapidly after the initial hour [136], in addition, it can cause a long-term impact among patients with pre-existing coronary heart disease. One very large study was conducted in the United States to assess the association of recreational marijuana use with the incidence of acute myocardial infarction. The study involved 2,451,933 patients with acute myocardial infarction aged 11–70 years old from 2010 to 2014. The study found that the lifetime myocardial infarction (ACI) odds were increased by up to 8% in cannabis users. However, the overall odds of marijuana-related mortality were not significantly increased in recreational users presenting with acute myocardial infarction [137,138].

A large study was conducted on 1913 myocardial infarction survivors in United States hospitals. The study found that cannabis use is associated with an increased risk of mortality in individuals with coronary heart disease; cannabis use was associated with a three-fold greater mortality after AMI. The increase in risk was graded with more frequent use, with adjusted hazard ratios of 2.5 for less than weekly use and 4.2 for weekly use [134]. A similar study was performed including 3886 survivors of myocardial infarction followed up for 18 years; the study found an apparent increased mortality rate of patients presenting with acute MI who were cannabis users, but the increase did not reach nominal statistical significance [139]. 

Recently, cannabis use is implicated as a risk factor for Takotsubo cardiomyopathy, characterized by transient left ventricular wall apical ballooning that leads to temporary left ventricular dysfunction. Nogi et al. reported a mid-ventricular variant takotsubo cardiomyopathy case associated with cannabinoid hyperemesis syndrome (recurrent episodes of severe nausea and vomiting, and colicky abdominal pain associated with long-term, heavy cannabis use) in a long-term marijuana user [140]. Khalid et al. reported a case of Takotsubo cardiomyopathy associated with a urine drug screen positive for THC [141]. A study performed by Alliu et al. to investigate the association of cannabis and Takotsubo cardiomyopathy concluded that there is a significant association between non-dependent cannabis use and increased odds of Takotsubo cardiomyopathy [142].

There are two reports of teenage boys diagnosed with cannabis-induced myocarditis [143,144]. One of them is a 16-year-old teenager who was diagnosed with acute left heart failure secondary to acute myocarditis due to cannabis abuse. The patient required support by a left ventricular assist device for 96 days until his cardiac function was fully recovered [144]. A case of pediatric death after exposure to cannabis was reported in 2017. The death was secondary to myocarditis in an 11-month-old male who was confirmed to be exposed to cannabis. The patient suffered from a seizure which was followed by CNS depression, and developed cardiac arrest and consequently has died [145]. Based on these cases, it is recommended to include cannabis exposure in the differential diagnosis of young patients presenting with myocarditis [143,145,146].

### 4.2. Cognitive, Psychiatric, and Psychomotor Effects

Activation of the CB1 receptor can lead to central side effects, such as ataxia and catalepsy [147]. Binding of THC to CB1 receptors can affect perception, memory, and movement, as a result of selective adenylate cyclase activity inhibition consequent to CB1 activation, and may cause dysphoria and psychotomimetic effects. 

**Figure 5:**
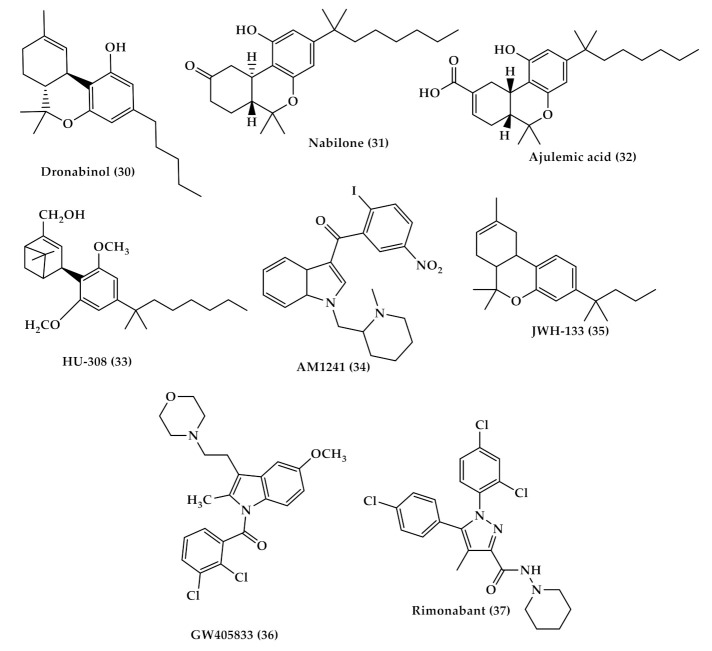
Chemical structures of the analgesic cannabinoids; dronabinol (**30**), nabilone (**31**), ajulemic acid (AJA (**32**)), HU-308 (**33**), AM1241 (**34**), JWH-133 (**35**), GW405833 (**36**) and rimonabant (**37**) a CB1 receptor inverse agonist that reduce appetite.

Cannabis use is linked to cognitive impairment; short-term memory impairment is a well-established effect of acute cannabis intoxication [148]. The memory impairment after cannabis use is thought to be attributed to THC’s effect on hippocampal CB1 receptors; the hippocampus is a region in the brain implicated in certain forms of learning and memory and is dense with CB1 receptors [149]. THC and CB1 agonists reduce presynaptic neurotransmitter release and consequently disrupt synaptic long-term plasticity in the hippocampus [150]. In a study performed on rodent models, rimonabant (37) (Figure 5), a CB1 receptor antagonist, was administered intrahippocampally followed by systemic administration of THC. It was found that THC’s memory deficits were reversed by rimonabant. These findings support the notion that hippocampal CB1 receptors mediate the memory deficit effect of THC [151]. Moreover, Marsicano et al. showed that CB1 knockout mice exhibited impaired short- and long-term extinction in tests of auditory fear conditioning, reduced forgetting, unaffected memory acquisition and consolidation on memory tests [152].

Although in comparison with alcohol use, long-term use of cannabis is not associated with severe cognitive impairment [153], memory problems are frequently associated with short and long-term cannabis use, such as subtle memory and attention impairment, as well as impaired ability to integrate and organize complex information. Cannabis-related cognitive impairment increases with the increased duration of use [112]. A study by Herning et al. found that heavy cannabis users showed a significant increase in cerebrovascular resistance and systolic blood flow velocity compared to control subjects; this increase persisted after a month of monitored abstinence, so the authors suggested that cannabis-related cognitive deficit is partially caused by increased cerebrovascular resistance and decreased blood perfusion to the brain [154].

Other central effects of cannabis include disruption of psychomotor performance, motor function, and reaction time, effects that are proposed to result from the memory lapse. In fact, acute administration of cannabis is associated with an initial stage of excitement and psychomotor agitation, followed by a state of dysarthria, ataxia, physical inertia, and general incoordination [112]. The psychomotor impairment effect of cannabis is additive to alcohol’s effect; this has made cannabis a major risk for road accidents [155,156]. Cannabis also is linked to increased interpersonal violence [157], an effect that can be attributable to acute psychomotor agitation. Additionally, a 12-year forensic investigation of deceased illicit drug users found that cannabis use was associated with the most violent suicide and death means, particularly severe motor vehicle accidents [158]. Some research studies showed a positive association between cannabis use and suicide attempts, but other investigations denied this association. Price et al. found that there is no evidence of an association between cannabis use and suicide risk after controlling for many variables such as life circumstances and mental health in childhood and early adolescence [153]. A study by Naji et al. showed that there is not a specific association and observed that the heaviness of cannabis use may have a very modest association with suicide attempts in adults. On the contrary, the impact of cannabis use can differ in people with psychiatric disorders, there being a significant risk factor for suicide attempts in psychiatric patients, such as unemployed and mood-disturbed people [154]. A new study by Fontanella et al. showed that cannabis use disorder (CUD) increases the risk of self-harm and death from involuntary or voluntary overdose in young people with mood disorders [155]. These findings should serve as information for healthcare professionals and policy makers to support patients with mood or psychiatric disorders.

In general, the most common adverse psychiatric effect of cannabis is anxiety [156,159]. However, several studies showed a relation between cannabis use and the risk of acute toxic psychosis and schizophreniform spectrum disorders [3,160,161,162,163,164,165,166,167]. Low doses of THC (2.5–15mg) can affect memory, motor function, and other acute side effects, while large doses of THC (˃100mg) can produce chronic adverse effect such as delusions, disorientation, visual and auditory hallucinations, paranoid ideations, mania, and schizophreniform psychosis [156,168]. These reactions are usually dose-related and self-limiting, and they can last from a few days to weeks, depending on the potency of the preparation. Cannabis users with a family history of psychotic disorders are particularly vulnerable to the psychotic adverse effects of cannabis. Cannabis was found to precipitate psychosis in people with a family history of psychotic disorders [156]. There is sufficient evidence that the frequent use of cannabis by young people could increase the risk of developing psychotic disorders or having the first episode of psychosis earlier than usual in their life [168,169]. Cannabis also has been found to exacerbate pre-existing mental illness. Furthermore, long-term cannabis use is implicated in other mental disorders such as bipolar disorder [159,170,171,172] and depression [173,174].

### 4.3. Effects on the Respiratory System 

Cannabis (marijuana) is the most commonly smoked substance in the world after tobacco. Among the different ways of intaking cannabis, inhalation through the lungs is the most usual intake method, whether by cigarette smoking, pipes, or vaping using special devices. Compared with tobacco smoking, cannabis smoking is usually done with deeper inhalation and greater breath-holding time in order to achieve a higher absorption of THC. Effects of cannabis smoking on the lungs depend on the depth of inhalation and the duration of breath holding. The pattern of smoking cannabis results in greater deposition of substances with toxic potential in the lungs [175], and the physical dynamics of smoking were even proposed to cause lung disease other than cannabis itself [176]. As for the composition of cannabis smoke, it contains THC, the main psychoactive component, and other substances such as CBN, CBD, and a large mixture of compounds including volatile components such as ammonia, carbon monoxide, hydrocyanic acid, and nitrosamines, and tar components (phenols, naphthalene, benzanthracene, procarcinogenic benzopyrenes) [177]. Many of these compounds are bronchial irritants, mutagens, and carcinogens [178,179].

Chronic smoking of cannabis is associated with bronchitis, emphysema, and squamous metaplasia of the tracheobronchial epithelium. These symptoms are more frequent in cannabis-only smokers than in tobacco-only smokers, and they are even higher in those who smoke both cannabis and tobacco [180]. Other described pulmonary complications of cannabis include lung bullae and cystic lung disease, signs of the destruction of lung tissue, decreased lung density with increased lung volumes, and secondary pneumothorax because of bullous rupture [176,181,182,183,184]. Cannabis smoking is also associated with lung cancer, as the smoke contains a number of potent carcinogenic compounds [185,186,187,188,189]. Low doses of THC were found to accelerate the proliferation of lung carcinoma cells in vivo [190]. Moreover, several case reports have suggested a link between cannabis smoking and aero digestive tract (mouth, tongue, esophagus) cancer [179].

For further understanding the relationship between cannabis smoking and pulmonary function, Tetrault et al. conducted a systematic review of the literature and selected 34 relevant publications examining short-term and long-term cannabis smoking and pulmonary function and respiratory complications. The study concluded that short-term cannabis smoking is associated with acute bronchodilation. In fact, for many years, marijuana was used as an alternative medicine to treat asthma symptoms due to its mild bronchodilation. while in the longer term, cannabis smoking is associated with increased respiratory symptoms of coughing, phlegm, and wheezing, suggestive of obstructive lung disease [191]. These findings were supported by the conclusion of a systemic review and meta-analysis of 22 studies, in which low-quality evidence that associates cannabis smoking with coughing, sputum production, and wheezing was found [192]. Additionally, a patient-centered observational study was conducted on 8932 patients with cannabis use, or at least two cannabis positive urine drug screens, and matched with non-cannabis-using patients; the results show that cannabis use was associated with a higher risk for pneumonia, asthma, and COPD, regardless of tobacco use disorder state [193]. Some in vitro and animal studies have suggested that cannabis results in impaired bactericidal activity of lung alveolar macrophages and consequently a depression of the intrapulmonary antibacterial defense systems [194,195]. Despite that, there is no clear evidence that cannabis smoke causes significant immunological damage in humans.

### 4.4. Effects on the Hormonal System and Fertility

Men and women of reproductive age are the most prevalent users of cannabis, and thus cannabis’ impact on the fertility and reproduction system is of special importance. Cannabinoids, including THC, were found to exert anti-androgenic effect by binding to androgen receptors and acting on the hypothalamus-pituitary-adrenal (HPA) axis [196]. Animal studies have shown that cannabis influences several endocrine processes affecting sexual hormones as well as other hormones like melatonin and growth hormone [196,197,198]. It was demonstrated by animal models that cannabis is related to testicular atrophy, reduced libido, and sexual function [196,199]. However, until now, such effects have not been observed in human studies [199].

Cannabinoid receptors are expressed in human sperm [200]; this suggests that sperm can be directly affected by alterations in the endocannabinoid balance. Research in male humans found evidence that cannabis has a role in reducing sperm count and concentration, decreasing sperm motility and viability, inducing abnormalities in sperm morphology, and inhibiting fertilizing capacity [194,201].

In addition, another research study demonstrated changes in sexual hormones upon using cannabis. While cannabis’ effect on testosterone levels is largely undetermined, luteinizing hormone (LH) levels appear to be lowered and levels of the follicle-stimulating hormone are unchanged (except in heavy chronic use cases) [199]. The role of follicle-stimulating hormone (FSH) is known in supporting developing spermatozoa by stimulating Sertoli cells, while LH stimulates testosterone production from Leydig cells [202]; lowered levels of FSH and LH by THC can cause reduced testosterone production by the Leydig cells [203]. Moreover, chronic cannabis use may increase prolactin concentrations in men, which may lead to gynecomastia [204].

Cannabis’ effect on female reproduction has not been fully studied. A study was conducted by Somenath Ghosh on female mice which were given different concentrations of cannabis extract orally by micro-pipette to study the effect of canna-bis on female reproductive system. The results show that chronic cannabis exposure induced oxidative stress and impairment in the female mouse reproductive system which is characterized by a significant decrease in ovarian and uterine weight [205]. Another study was conducted on healthy adult females and the results show that cannabis smoking causes an acute decrease in prolactin concentrations, but prolonged use may increase the hormone concentrations, leading to galactorrhea [204]. Cannabis can reduce estrogen and progesterone production by disrupting the hypothalamic release of gonadotropin-releasing hormone (GnRH), which may lead to an ovulatory menstrual cycle and reduced female fertility [205]. Cannabis inhalation during the luteal phase of the menstrual cycle may result in transient suppression of levels of prolactin and LH [206]. Nevertheless, there is no conclusive evidence of cannabis’ effect on menstruation or on levels of estrogens, progesterone, testosterone, prolactin, LH, or FSH in women [207,208].

In general, cannabis-related endocrine changes may not be significant in adults, but they may be of high importance in prepubescent males and females, in whom cannabis may suppress sexual maturation [204,209]. Several animal studies showed that cannabis (specifically exposure to THC) can lead to pubertal delay and affect pubertal maturation [210]. However, evidence in humans is limited except for a case of a 16-year-old youth who showed delayed puberty and low testosterone levels with heavy cannabis smoking, and after discontinuation of cannabis smoking, his testosterone levels increased and pubertal development advanced [211]. Hence, an urgent understanding of the effect of cannabis on pubertal timing and tempo in children is needed. A systematic review was conducted to evaluate the effects of cannabis use on puberty in humans. The results show that there are no existing articles that relate cannabis use with the delay in puberty in children and highlight the importance of this issue for future investigation [210].

### 4.5. Maternal Cannabis Exposure and Infant Outcome

Cannabis use among pregnant women is frequent. International studies estimated that 4.5–7% of all pregnancies are exposed to cannabis [212,213,214], and cannabis was the most common illegal drug among pregnant women in western countries [214,215,216]. Evaluation of maternal cannabis use’s effects on fetal and infant outcomes is difficult, as cannabis use is not always reported, and cannabis is usually consumed with other drugs, especially alcohol, tobacco, and caffeine.

THC and other cannabinoids cross the placenta rapidly and enter the embryo [217,218], and they are secreted in breast milk. There is no evidence of fetal malformations, but accumulating evidence from human and animal studies indicates that maternal cannabis use during pregnancy can alter fetal development and infant outcomes. Chronic maternal cannabis use is associated with reduced weight gain and low body weight at birth, which is the most noticed effect related to maternal cannabis exposure. Roncero et al. [213] conducted a systemic review of the literature on cannabis use during pregnancy and fetal outcomes, and they found that maternal cannabis use may be associated with developmental abnormalities in general, development of mental disorders such as depression and attention deficit hyperactivity disorder (ADHD), and changes in brain chemistry, which was observed in both humans and animals [213]. Many developmental defects related to prenatal cannabis exposure were identified by human longitudinal studies of in utero cannabis-exposed offspring. Defects in cognition, executive functioning, and visuospatial working memory, and lower school-age intellectual development were identified to be related to in utero cannabis exposure. In utero exposure to cannabis was associated with deficits in tasks requiring visual memory, analysis, and integration and with impulse control in tasks requiring sustained attention [219,220,221,222,223]. Cross-sectional retrospective studies have linked maternal cannabis use to impaired memory, impulse control, quantitative reasoning, problem-solving, and verbal development in children aged 1–11 years old [224], as well as alterations in emotional reactivity, neurobehavioral performance, neurophysiological integrity, increased tremors and startlings in infants [225,226,227].

A 17-year period study was conducted on infants and fetuses with birth defects to investigate the relation between cannabis use during pregnancy and birth defects. Investigation showed that birth defects associated with prenatal cannabis use affect the cardiovascular system, the gastrointestinal system, and the central nervous system [228]. In addition, a retrospective case-control study of 204 pairs found an 11-fold risk of developing non-lymphoblastic leukemia in the offspring of mothers who had been exposed to marijuana within a year before pregnancy or during pregnancy [229].

Huizink presented an overview of studies on prenatal cannabis exposure in humans in which findings on fetal development, birth outcomes, neonatal and infant behavior, and cognitive development were discussed. It was concluded that there is evidence that fetal development is affected by maternal cannabis use during pregnancy, while evidence is inconsistent on the effects on infant behavior and cognition [230].

Studies on animal models of prenatal cannabis exposure have supported the findings of altered cognitive, emotional, social, and motor aspects in offspring [231,232,233]. Rodents prenatally exposed to isolated THC or WIN 55,212-2 (a CB1 receptor agonist) have shown lasting changes in epigenetic regulation, synaptic plasticity, and dopamine neuron signaling [234,235,236,237]. However, synthetic cannabinoid or THC injections cannot be considered a good representative of whole-plant cannabis, which contains a large number of pharmacologically unique phytocannabinoids [12,238]. A recent study was conducted to assess the neurodevelopmental effects of maternal cannabis vapor exposure on offspring of rats by administering whole-plant cannabis extract vapor. In this study, a new approach of mimicking the intrapulmonary administration route was applied taking advantage of ‘e-cigarette’ technology to deliver vaporized extracts of whole-plant cannabis, to attain relevant concentrations in plasma and brain tissue. The study found that prenatal cannabis vapor exposure resulted in long-lasting effects on the behavioral profile that extend into adulthood, and the effects indicated an increase in emotional reactivity, alterations in social play behavior, and behavioral flexibility [239].

Cannabis use among pregnant women is not extensively researched. Additional research is needed to detect and better understand the effects of maternal cannabis use on fetal development and offspring. Early detection, alerting, and education of women in childbearing years on the effects of cannabis use during pregnancy is necessary to minimize possible harm. Moreover, it is also important for healthcare professionals to have good scientific knowledge and to be well trained in this field.

### 4.6. Cannabis-Tolerance and Dependence

Increasing cannabis use due to growing legalization in different countries world-wide led to the development of cannabinoid abuse disorders and adverse effects [240]. Cannabis use disorder is defined according to the Diagnostic and Statistical Manual of Mental Disorders (DSM–5) as a pathological problematic pattern that leads to control and social impairment, physiological adaptation, and tolerance [241,242]. Long-term and heavy chronic use of cannabinoids can cause addiction, especially when started in adolescence, and tolerance, which results in physical dependence that leads to withdrawal syndrome when stopping drug use [240]. Cannabis withdrawal symptoms can start after 1 or 2 days of cessation and last for one to two weeks, and the symptoms include irritability, depression, decreased appetite, anger, and difficulty sleeping, which is similar to tobacco withdrawal syndrome [242,243]. These symptoms may vary among individuals and depends on the amount and potency of cannabis use prior to cessation; usually mild to moderate symptoms are treated in an outpatient detoxification setting, but severe symptoms need inpatient care [244]. Different approaches are now considered for the treatment of cannabis withdrawal symptoms. Recently, cannabidiol was considered as a potential treatment for cannabis withdrawal symptoms due to its safety and tolerability with few side effects and broad range of pharmacological effects that include the inhibition of hydrolysis and reuptake of endocannabinoids, in addition to its ability to interact with the effects of THC. A phase 2a trial was conducted to determine the efficacious and safe doses of cannabidiol, which were 400 and 800 mg, to reduce cannabis use [245]. Nicotin patches (NP) at 7 mg dose were also studied for their potential to reduce withdrawal symptoms in cannabis-dependent individuals who are not heavy users or not nicotine dependent, and the results showed that NP has the ability to reduce withdrawal negative effect in individuals with cannabis use disorder [246]. Moreover, exogenous progesterone was suggested as a therapy in women who suffer acute cannabis withdrawal symptoms due to its noticed effect in reducing cannabis craving. Large size and longer duration studies are needed to support the role of exogenous progesterone in the assessment of cannabis withdrawal symptoms [247]. The effects of other drugs on reducing cannabis withdrawal symptoms were also investigated, such as nabiximols, nefazodone, lofexidine, and oral THC; the results show their ability in the reduction in anxiety, sleep disorders, craving, and depressed mood, but they were associated with negative side effects that worsen withdrawal symptoms, such as irritability [248,249]. More studies need to be conducted in order to better understand cannabis dependence. Until now, there are no approved drugs for the treatment of cannabis dependence, and psychotherapeutic techniques are the main therapy [250].

### 4.7. Cannabinoids Drug Interactions

Cannabinoids such as THC, CBD, and CBN are extensively metabolized by cytochrome P450 in the liver and intestines. THC and CBN biotransformation occurs mainly due to CYP2C9 and CYP3A4, while CBD biotransformation occurs due to CYP3A4, and may undergo direct conjugation via UDP-glucuronosyltransferase (UGT) enzymes. Several drugs can affect cannabinoid metabolism, and at the same time, cannabinoids can affect the metabolism of other drugs [251,252]. CYP3A4 inhibitors such as ketoconazole, macrolide, verapamil, and augmentin can increase THC and CBD concentrations to double [253]. CYP2C9 inhibitors such as cotrimoxazole, fluoxetine and amiodarone can also increase THC concentration and its psychoactive effects. On the other hand, CBD inhibits CYP2C19 and can increase the level of clobazam to threefold, increase bleeding when combined with warfarin, and increase tacrolimus levels by three-fold by inhibiting CYP3A4/5 [254,255]. Additive effects can also occur when combining cannabis with other drugs such as (i) sympathomimetics, which can result in tachycardia, hypotension and hypertension; (ii) CNS depressants, e.g., alcohol, or muscle relaxants and opioids, provoking drowsiness and ataxia; and (iii) anticholinergic, which cause tachycardia and confusion [253,254]. In the case of smoked cannabis, drug clearance can be increased with regular cannabis use, i.e., more than two cigarettes per week. Smoked cannabis increases the clearance of several drugs metabolized by CYP1A2 such as theophylline, which can be increased to 40% in addition to clozapine and olanzapine [248,253].

## 5. Legalization of Cannabis

Cannabis legalization is rising in countries worldwide and the opinions about its use are split into supporters who believe that cannabis legalization can improve public health, stimulate the economy, and reduce criminal justice expenditure, while critics believe that legalization will increase cannabis use, which may affect health and safety, lower the educational achievement in teens, and increase crime [256].

To date, the FDA has only approved one cannabis-derived drug product (Epidiolex (cannabidiol)) and three synthetic cannabis (dronabinol (Marinol and Syndros) and nabilone (Cesamet)) products, and did not approve any other cannabis or its derivatives. Cannabis is classified in Schedule I by the FDA, where substances that have high potential for abuse are restrictively categorized [5,257]. Cannabis being in Schedule I increases the complexity for research studies, because all researchers must follow Schedule I research registrations. In addition, cannabis is a controlled substance and researchers must obtain it from registered cultivators with the Drug Enforcement Administration (DEA) and licenses from the state-controlled drug authority [257,258]. Medical use of cannabis and cannabinoids must be supervised by medical practitioners and dispensed by prescription where the drug should be used only when necessary [259]. Although cannabis is federally illegal in the US, nine US states have legalized adult recreational use of cannabis since 2012. Furthermore, in 1999, Canada allowed the use of cannabis for medical issues, and in 2018, it decriminalized and legalized recreational cannabis use. The UK also legalized medical cannabis in 2019, in addition to other countries worldwide. Changes in legalization laws and the liberalization of cannabis increase the need for more research to understand the cannabis plant and its effects, which may be available in the future [260,261,262,263].

## 6. Conclusions

Cannabis has been used since ancient times to the modern times for recreational and medicinal purposes. In most countries, cannabis is considered an illicit drug, although there are several drugs based on cannabinoids present in cannabis. Cannabis is a very rich source of chemical compounds, the majority of which are called phytocannabinoids. These entities have a variety of physiological and often psychoactive effects; they exert their effects mainly by binding to endogenous cannabinoid receptors, CB1 and CB2. The most prevalent phytocannabinoids are THC, which is a compound responsible for the psychotropic effects of cannabis and generally is the main constituent responsible for the toxic effects related to cannabis, and the non-intoxicating compound, CBD.

Cannabis has potential therapeutic properties, and its preparations have been used as traditional remedies for treating pain and emesis. Synthetic cannabinoids, which are analogs of phytocannabinoids, are used clinically as analgesics, antiemetic, and appetite stimulants.

Significant cannabis toxicity is uncommon in adults, but it can cause a wide range of toxic effects acutely and in the long term. Acute poisoning is characterized by neurological symptoms (dizziness, drowsiness, ataxia, seizures, hypotonia, stupor, and coma), ocular symptoms (mydriasis and conjunctival hyperemia), gastrointestinal symptoms (nausea, vomiting, and thirst), and cardiovascular features (tachycardia and arterial hypertension). Cannabis toxicity is more significant in children and in users with preexisting cardiac, pulmonary, or mental diseases. Most toxic effects arise from CB1 receptor activation. Cannabis use is related to major adverse cardiovascular events, cognitive impairment, psychomotor performance disruption, exacerbation of the pre-established mental disease. Smoking cannabis is linked to bronchitis and reduced pulmonary function, and it increases the risk of respiratory disease and lung cancer. Cannabinoids may result in changes in sex hormones in males and females. Maternal cannabis use during pregnancy affects fetal development, and it may affect the infant cognition and behavior.

Between the promising therapeutic advantages, the high tendency of abuse, and the safety concerns, additional research efforts are still needed to better understand the cannabinoid interactions within the human body and to explore the potential medical applications of cannabis.

## Data Availability

Not applicable.
